# Identification of Immune-Related Subtypes and Characterization of Tumor Microenvironment Infiltration in Bladder Cancer

**DOI:** 10.3389/fcell.2021.723817

**Published:** 2021-08-31

**Authors:** Mengjia Huang, Lin Liu, Junkai Zhu, Tong Jin, Yi Chen, Li Xu, Wenxuan Cheng, Xinjia Ruan, Liwen Su, Jialin Meng, Xiaofan Lu, Fangrong Yan

**Affiliations:** ^1^State Key Laboratory of Natural Medicines, Research Center of Biostatistics and Computational Pharmacy, China Pharmaceutical University, Nanjing, China; ^2^Department of Urology, The First Affiliated Hospital of Anhui Medical University, Hefei, China; ^3^Anhui Province Key Laboratory of Genitourinary Diseases, Institute of Urology, Anhui Medical University, Hefei, China

**Keywords:** ICI, immune subtype, tumor microenvironment, bladder cancer, immunotherapy

## Abstract

Tumors are closely related to the tumor microenvironment (TME). The complex interaction between tumor cells and the TME plays an indisputable role in tumor development. Tumor cells can affect the TME, promote tumor angiogenesis and induce immune tolerance by releasing cell signaling molecules. Immune cell infiltration (ICI) in the TME can affect the prognosis of patients with bladder cancer. However, the pattern of ICI of the TME in bladder cancer has not yet been elucidated. Herein, we identified three distinct ICI subtypes based on the TME immune infiltration pattern of 584 bladder cancer patients using the ESTIMATE and CIBERSORT algorithms. Then, we identified three gene clusters based on the differentially expressed genes (DEGs) between the three ICI subtypes. In addition, the ICI score was determined using single sample gene set enrichment analysis (ssGSEA). The results suggested that patients in the high ICI score subgroup had a favorable prognosis and higher expression of checkpoint-related and immune activity-related genes. The high ICI score subgroup was also linked to increased tumor mutation burden (TMB) and neoantigen burden. A cohort treated with anti-PD-L1 immunotherapy confirmed the therapeutic advantage and clinical benefit of patients with higher ICI scores. In the end, our study also shows that the ICI score represents an effective prognostic predictor for evaluating the response to immunotherapy. In conclusion, our study deepened the understanding of the TME, and it provides new ideas for improving patients’ response to immunotherapy and promoting individualized tumor immunotherapy in the future.

## Introduction

Bladder cancer is the most common malignant tumor of the urinary system. It accounts for the highest incidence of genitourinary tumors in China and is the second most common genitourinary malignancy in the United States (Kaufman et al., 2009). Bladder cancer can occur at any age, even in children, and its incidence increases with age and in those aged 50–70 years old. The incidence of bladder cancer in males is three to four times higher than that in females (Kaufman et al., 2009; Sanli et al., 2017). According to the histological classification of urinary tract tumors in the 2004 WHO “Pathology and Genetics of Tumors in the Urological System and Male Reproduction Organ,” the pathological types of bladder cancer include bladder urothelial carcinoma, bladder squamous cell carcinoma and bladder adenocarcinoma. Other rare disease subtypes include bladder clear cell carcinoma, bladder small cell carcinoma and bladder carcinoid. Among them, bladder urothelial carcinoma is the most common, accounting for more than 90% of the total number of bladder cancer patients. Urothelial carcinoma of the bladder can be divided into non-muscle invasive urothelial carcinoma (NMIBC) and muscle invasive urothelial carcinoma (MIBC), and approximately 75% of newly diagnosed patients have non-muscle invasive bladder cancer with 25% having muscle invasive bladder cancer (Kaufman et al., 2009; Sanli et al., 2017). Most patients with non-muscle invasive urothelial carcinoma receive transurethral resection of bladder tumors and bladder perfusion therapy to prevent recurrence postoperatively. Total cystectomy is often used in patients with muscle invasive urothelial carcinoma, squamous cell carcinoma and adenocarcinoma of the bladder, and partial cystectomy may be used in some patients. Neoadjuvant chemotherapy combined with surgery is also recommended for patients with muscle invasive urothelial carcinoma. Metastatic bladder cancer is primarily treated with chemotherapy. Approximately 70% of patients relapse after transurethral resection, and Bacillus Calmette-Guerin (BCG) or chemotherapy can reduce the recurrence rate to 25–40%.

Immunotherapy is a treatment method that artificially enhances or suppresses the immune function of the body to treat diseases by harnessing the immune state of the body, which is low or hyperactive. Tumor immunotherapy aims to activate the human immune system, kill tumor cells and tissues through autoimmune function, and restore the normal antitumor immune response of the body by restarting and maintaining the tumor-immune cycle to control and eliminate tumors. It includes monoclonal antibody immune checkpoint inhibitors, therapeutic antibodies, cancer vaccines, cell therapy, small molecule inhibitors and so on. Immunotherapy has evolved in recent years and has been proven to treat a variety of cancers, including melanoma, non-small cell lung cancer, kidney cancer and prostate cancer (Del Paggio, 2018). For example, ipilimumab improves survival in melanoma patients (Hodi et al., 2010; Schadendorf et al., 2015). Immunotherapy strategies for bladder cancer include intravesical administration of BCG (Alexandroff et al., 1999) and immune checkpoint inhibitors. However, studies have shown that immunotherapy is effective in only a small number of patients (Julie and Scott, 2012; Christofi et al., 2019). Therefore, new therapeutic markers are needed to identify the subgroup of patients who are suitable for immunotherapy.

The TME has been recognized as an important component of malignant tumor tissues and plays a mixed role in tumor progression, metastasis, treatment resistance and disease recurrence (Runa et al., 2017). The TME refers to the surrounding microenvironment of tumor cells, including blood vessels, immune cells, fibroblasts, bone marrow-derived inflammatory cells, various signaling molecules and the extracellular matrix (ECM). The complex interaction between tumor cells and the TME plays an indisputable role in tumor development (Pottier et al., 2015). Tumors are closely related to the TME. Tumors can affect their microenvironment, promote tumor angiogenesis and induce immune tolerance by releasing cell signaling molecules (Kerkar and Restifo, 2012). Tumor cells promote the growth and development of tumors by changing and maintaining the conditions of their survival and development through autocrine and paracrine mechanisms. Immune cells in the microenvironment include adaptive immunity, T lymphocytes, dendritic cells and accidental B cells, innate immunity, macrophages, polymorphonuclear leukocytes, and natural killer cells, which can influence tumor cell growth and development (Whiteside, 2008). Therefore, it is possible to identify different immune phenotypes by analyzing the heterogeneity and complexity of the TME, improving the ability to guide and predict responses to immunotherapy.

In this study, we identified three distinct immune ICI subtypes based on the infiltration patterns of 22 immune cells obtained using the CIBERSORT algorithm and the immune score and stroma score computed by the ESTIMATE algorithm of 584 bladder cancer tumor samples. Samples were further divided into three gene clusters based on DEGs identified based on three ICI subtypes. In addition, we established an ICI score to characterize the immune landscape of bladder cancer, which can accurately predict patients’ outcomes and responses to immunotherapy. Finally, the ICI score was validated in an independent cohort. The results indicated that the ICI score can be used as an effective prognostic predictor of immunotherapy. The evaluation of ICI patterns in larger samples may provide new directions for strategies of immunotherapy in bladder cancer.

## Materials and Methods

### Study Cohort and Data Pre-processing

Bladder cancer data were obtained from five publicly available datasets, TCGA-BLCA, GSE13507, GSE31684, E-MTAB-1803, and a validation cohort (GSE93527). The RNA-Seq data (fragments per kilobase million value) of the TCGA-BLCA cohort were downloaded using the R package “TCGAbiolinks,” and the FPKM values were transformed into TPM values. Four hundred and fourteen samples of bladder cancer were obtained by considering only the mRNA-encoding protein. Clinical and survival information of TCGA-BLCA was extracted from pan-cancer data, including age, sex, stage, etc. Only overall survival (OS) was considered. Tumor samples lacking clinical information were excluded, and samples with 0 OS were also removed. Common samples of expression profile data and survival information were extracted, and 406 bladder cancer samples were obtained from the TCGA-BLCA cohort. The copy number variation data were downloaded from Fire Browse^1^. Mutation data were downloaded from cBioPortal^2^. The number of predicted neoantigens of TCGA-BLCA samples was obtained from a published article (Rooney et al., 2015). The GSE13507 cohort and GSE93527 cohort were downloaded from the Gene Expression Omnibus database^3^ on the Illumina platform. The microarray datasets (GSE31684 and E-MTAB-1803) were downloaded from the Array Express database^4^ on the Affymetrix platform. Samples with non-muscle invasive bladder cancer (NMIBC) samples were excluded, and only patients with muscle invasive bladder cancer (MIBC) were retained. Similarly, tumor samples lacking clinical information and samples with 0 OS were removed. Four datasets were combined, and the “Combat” algorithm was used to eliminate the batch effect (Supplementary Figure 2). In the end, a total of 584 bladder cancer samples were obtained for subsequent analysis. Detailed information on 584 bladder cancer patients is shown in Supplementary Table 1. The validation cohort, Imvigor210, was obtained from the R package “Imvigor210coreBiologies,” including survival outcomes, response results and expression profiles of patients who received immunotherapy (Mariathasan et al., 2018). The functional annotation gene set (h.all.v7.2.symbol) was downloaded from the MSigDB database^5^. The data of cell lines in bladder cancer were downloaded from DepMap^6^.

### Unsupervised Clustering Analysis

The level of ICI in bladder cancer was quantified by the CIBERSORT algorithm using the LM22 gene signature (Newman et al., 2015). The immune score and stroma score of each sample were calculated using the ESTIMATE algorithm (Yoshihara et al., 2013). Unsupervised clustering was applied to classify patients into distinct ICI subtypes according to the above 24 signatures. Consensus clustering was used to determine the number of clusters and stability using the R package “ConsensusClusterPlus” (Wilkerson and Hayes, 2010). We repeated 1,000 times (each using 90% of the samples) to guarantee the stability of clustering. The optimal cluster number was determined by the clustering score for the cumulative distribution function (CDF) curve and the relative changes in the area under the CDF curve.

### Identification of Differentially Expressed Genes

To identify ICI pattern-related genes, patients with bladder cancer were classified into three distinct ICI subtypes. The R package “limma” was applied to determine DEGs between different ICI subtypes (Ritchie et al., 2015). The significance criteria for determining DEGs were set as adjusted *p* < 0.05 and fold change cut-off of 1.65.

### Generation of ICI Score

To quantify ICI patterns of individual tumors, the ICI score was calculated. First, patients were classified into different groups using an unsupervised clustering method based on overlapping DEGs. We performed consensus clustering to determine the number of clusters that was repeated 500 times (each using 90% of the samples) of Spearman distance measurement using the PAM clustering method to enhance the stability of clustering. Second, we performed Pearson correlation analysis between DEGs and the cluster signature and obtained ICI gene signatures A and B, which were positively and negatively correlated with the cluster signature, respectively. Finally, we used the Boruta algorithm to reduce the dimension of ICI gene signatures. And then we used ssGSEA to calculate the signature scores (Barbie et al., 2009; Kursa and Rudnicki, 2010). ssGSEA was performed using the R package “GSVA.” We then defined the ICI score using a method similar to the gene expression grade index (GGI) (Sotiriou et al., 2006; Zeng et al., 2019):

$\mathrm{ICI}\mathrm{score}=\sum {\text{Score}}_{\text{A}}-\sum {\text{Score}}_{\text{B}}$

### Somatic Mutation Analysis

The mutation data of TCGA-BLCA tumor samples were downloaded from cBioPortal (see text footnote 2). Studies have shown that higher TMB and somatic mutation rates are correlated with stronger antitumor immunity (Rooney et al., 2015). To determine the relationship between somatic mutation and ICI score, we first classified patients into two subgroups, the high and low ICI score subgroups, and then used the R package “maftools” to identify driver genes in the high and low ICI score subgroups (Mayakonda et al., 2018). The top 25 driver genes with the highest mutation frequency were further analyzed.

### Copy Number Variation Analysis

The copy number variation data of TCGA-BLCA tumor samples were downloaded from Fire Browse (see text footnote 1). The Genomic Identification of Significant Targets in Cancer (GISTIC2.0) algorithm was utilized to classify the copy number variant genes with remarkable gains and losses (Mermel et al., 2011). The confidence level was set to 0.95, and other parameters were left at default settings (Luo et al., 2020; Yang et al., 2020).

### Collection of Genomic and Clinical Information With Immunotherapy

To further validate the predictive value of prognosis of the ICI score, we applied the ICI score to an independent anti-PD-L1 immunotherapy cohort, IMvigor210. The expression data and detailed clinical annotations of this cohort were obtained using the R package “IMvigor210CoreBiologies” that can be downloaded from http://research-pub.gene.com/IMvigor210CoreBiologies (Mariathasan et al., 2018). For gene expression data, the count value was also transformed into the TPM value with log_2_(TPM+1) for further analysis.

### Statistical Analysis

All statistical analyses were conducted using R version 4.0.2. The Kruskal–Wallis test was used for statistical comparison among more than two groups, and the Wilcoxon test was used for two group comparisons (Hazra and Gogtay, 2016). Kaplan–Meier curves were used to evaluate survival time in patients with bladder cancer. The OS probability was evaluated. The log-rank test was utilized to identify significant differences. The Wilcoxon test was used to analyze the correlation between the ICI score subgroups and somatic mutation frequency, and Pearson analysis was used to compute the correlation coefficient. All statistical *p*-values were two-sided, with *p* < 0.05 indicating statistical significance.

## Results

### Identification of ICI Subtypes in Bladder Cancer

**FIGURE 1:**
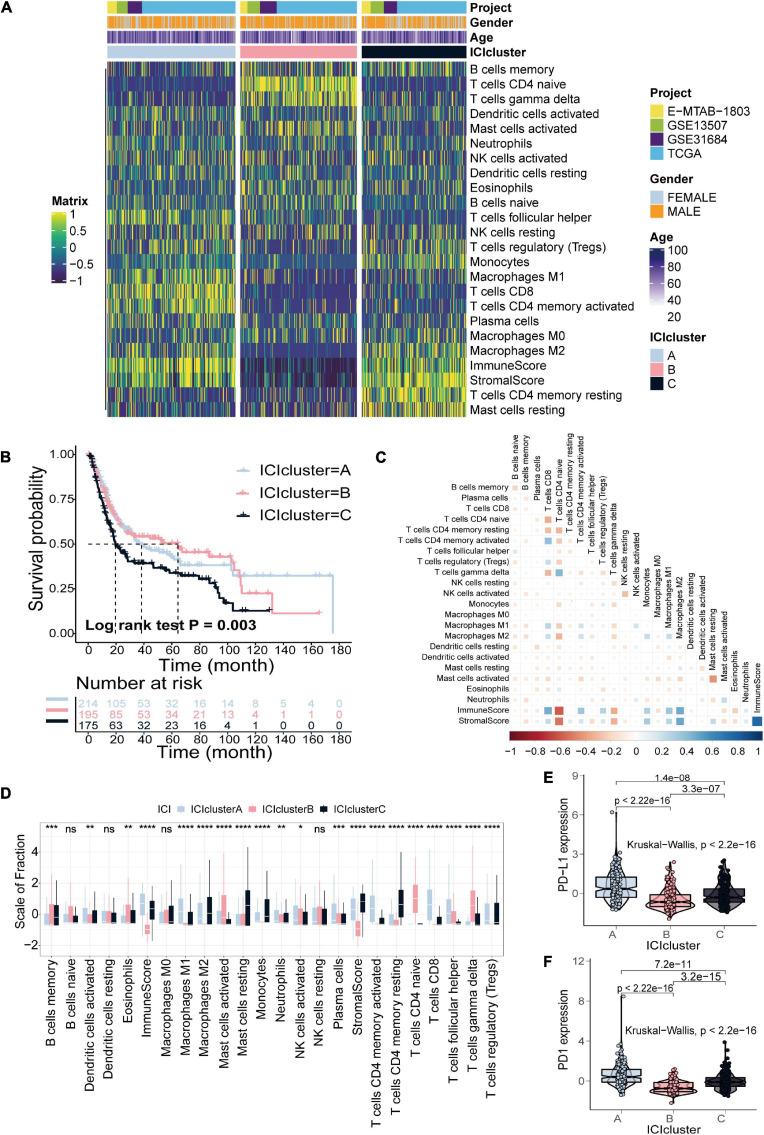
Identification of ICI subtypes in bladder cancer. **(A)** Consensus clustering of TIICs in four BLCA cohorts. **(B)** Kaplan–Meier curves for OS of all BLCA patients with ICI clusters (log-rank test, *p* = 0.003). **(C)** Cellular interaction of the TIICs types. **(D)** The fraction of TIICs, immune score and stromal score in three ICI clusters (Kruskal–Wallis test, *p < 0.05; **p < 0.01; ***p < 0.001; ****p < 0.0001). **(E,F)** The difference in PD-L1 **(E)** and PD-1 **(F)** expression among three ICI clusters (Kruskal–Wallis test, *p* < 2.2e-16).

The detailed workflow for ICI score construction is shown in Supplementary Figure 1. The abundance of 22 immune cells was estimated using the CIBERSORT algorithm, the enrichment scores of stromal cells and immune cells were estimated using the ESTIMATE algorithm, and 24 signatures were obtained for clustering analysis (Supplementary Table 2; Yoshihara et al., 2013; Newman et al., 2015). We identified three distinct ICI subtypes based on the above 24 signatures in 584 patients with bladder cancer, designated ICI clusters A–C (Figure 1A and Supplementary Figure 3). The OS curve of the three ICI subtypes was obtained using the Kaplan–Meier method (log-rank test, *p* = 0.003; Figure 1B). To visualize immune cell interactions in the TME, we also analyzed the correlations between 22 immune cells (Figure 1C). Additionally, we compared the composition of the TME of three distinct ICI subtypes to further elucidate differences among ICI subtypes. The infiltration levels of CD8+ T cells, activated natural killer cells, activated memory CD4+ T cells, follicular helper T cells and M1 macrophages were significantly higher in ICI cluster A. Patients in ICI cluster B were characterized by a significantly higher infiltration level of naive CD4+ T cells, gamma delta T cells and activated mast cells. ICI cluster C was marked by increased monocytes, M2 macrophages, resting memory CD4+ T cells and resting mast cells infiltration and had the poorest prognosis (Figure 1D). Finally, we analyzed and compared the expression levels of two important immune checkpoints of the ICI subtypes, PD-L1 and PD1. The results indicated that ICI cluster A exhibited the highest expression level of the two immune checkpoints (Figures 1E,F).

### Identification of Gene Subtypes Based on DEGs

**FIGURE 2:**
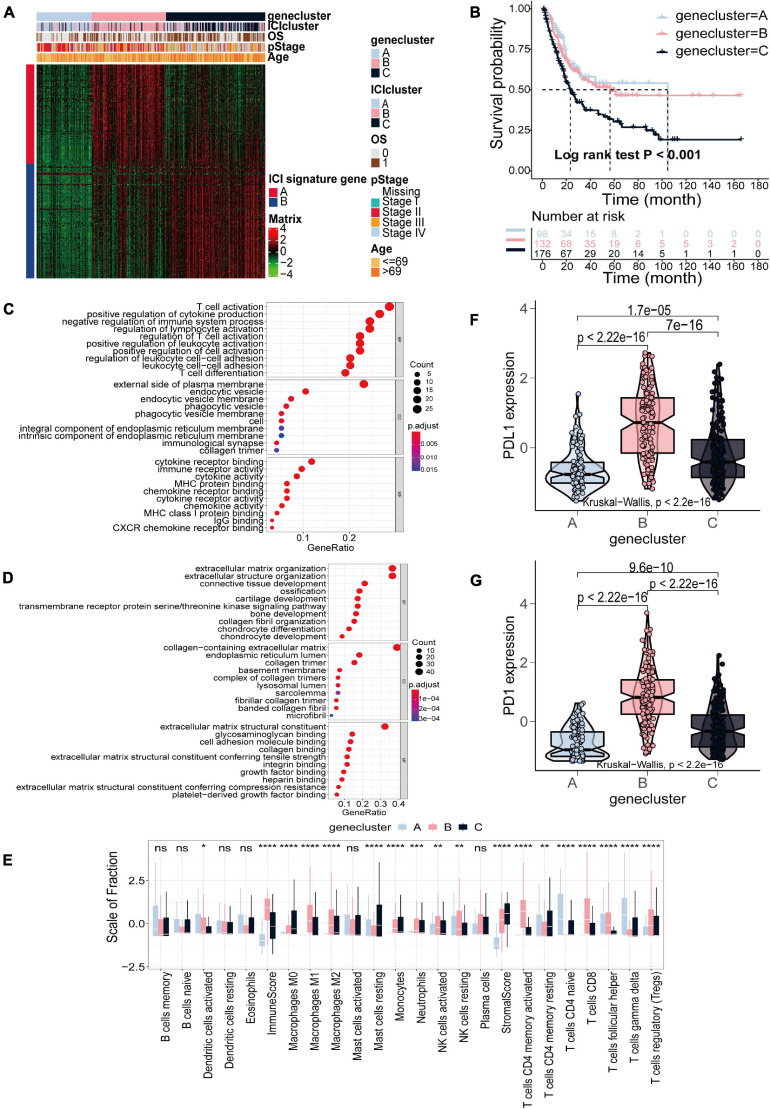
Identification of gene subtypes based on DEGs in TCGA-BLCA cohort. **(A)** Consensus clustering of DEGs among three ICI subtypes. **(B)** Kaplan–Meier curves for OS of the three gene clusters (log-rank test, *p* < 0.001). **(C,D)** GO enrichment analysis of the two ICI-related signature genes: ICI signature genes A **(C)** and ICI signature genes B **(D)**. **(E)** The fraction of TIICs, immune score and stromal score in three gene clusters (Kruskal–Wallis test, *p < 0.05; **p < 0.01; ***p < 0.001; ****p < 0.0001). **(F,G)** The difference in the expression of PD-L1 **(F)** and PD-1 **(G)** among three gene clusters (Kruskal–Wallis test, *p* < 2.2e-16).

Subsequent analysis was only based on the TCGA-BLCA cohort. To elucidate the difference in biological features in ICI subtypes, we performed differential analysis using the R package “limma” to determine transcriptome differences between ICI subtypes (Ritchie et al., 2015). Finally, 857 DEGs were identified (Supplementary Table 3). Then, 506 gene signatures A and 351 gene signatures B were identified by Pearson correlation analysis (Supplementary Table 4). At the same time, we used the Boruta algorithm to reduce the dimensions of gene signatures A and B, and 97 gene signatures A and 112 gene signatures B were finally obtained (Kursa and Rudnicki, 2010). Subsequently, unsupervised clustering was applied to classify patients into three subtypes based on 857 DEGs, gene clusters A–C (Figure 2A and Supplementary Figure 4). Gene cluster A exhibited lower expression of gene signatures A and gene signatures B. Gene cluster B had a higher expression level of gene signatures A and lower expression level of gene signatures B, and gene cluster C was the opposite with higher expression of gene signatures B and lower expression of gene signatures A (Figure 2A). The OS curve of the three gene subtypes was obtained using the Kaplan–Meier method (log-rank test, *p* < 0.001; Figure 2B). We found that patients in gene cluster C had the poorest prognosis. Gene ontology (GO) enrichment analysis was executed. The significantly enriched biological processes of gene signatures A and gene signatures B are summarized in Figures 2C,D, respectively. Detailed information on the enrichment analysis is shown in Supplementary Table 5. We also found that gene cluster A exhibited increased activated dendritic cells, naive CD4+ T cells, and gamma delta T cell infiltration. Gene cluster B, with the highest immune score, exhibited higher infiltration of M1 and M2 macrophages, natural killer cells, activated memory CD4+ T cells, CD8+ T cells and regulatory T cells. Gene cluster C displayed an escalated stroma score and higher infiltration of M0 macrophages and resting mast cells (Figure 2E). Similarly, analyzing the expression level of two immune checkpoints showed that gene cluster B had the highest expression of PD1 and PD-L1 (Figures 2F,G).

### Construction of the ICI Scores

**FIGURE 3:**
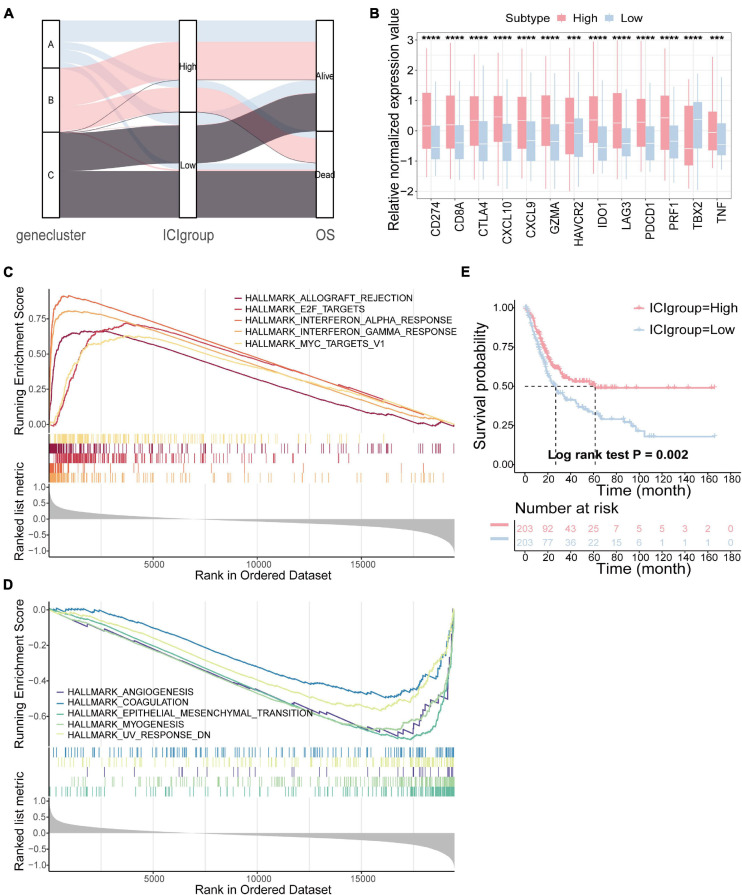
Construction of the ICI score. **(A)** Alluvial diagram of gene cluster distribution in groups with different ICI score and survival outcomes. **(B)** The difference in the expression of immune checkpoint-related genes (IDO1, CD274, HAVCR2, PDCD1, CTLA4, and LAG3) and immune activity-related genes (CD8A, CXCL10, CXCL9, GZMA, PRF1, TBX2, and TNF) between high and low ICI score subgroups. **(C,D)** The gene set enrichment analysis (GSEA) in high **(C)** and low ICI score subgroups **(D)**. **(E)** Kaplan–Meier curves for high and low ICI score groups in the TCGA-BLCA cohort (log-rank test, *p* = 0.002). ***p < 0.001; ****p < 0.0001.

To quantify the ICI patterns of individual tumors, the ICI score was constructed (Zhang et al., 2020). ssGSEA was used to calculate the score of gene signatures A and B, score A and score B, and then the prognostic signatures score was obtained. Patients were classified into two subgroups as high or low ICI score groups by the median score in the TCGA-BLCA cohort. The distribution of patients in three gene subtypes and two subgroups is shown in Figure 3A. We compared the expression levels of the immune checkpoint- and immune activity-related genes between the two groups to evaluate the immune activity and tolerance condition (Hugo et al., 2016; Ayers et al., 2017). We found significant overexpression of most immune checkpoint- and immune activity-related genes in the high ICI group, except TBX2 (Figure 3B). Gene set enrichment analysis (GSEA) was then executed (Figures 3C,D). Detailed information is provided in Supplementary Table 6. In addition, survival analysis revealed that the prognosis of patients in the high ICI score group was better than that in the low ICI score group (log-rank test, *p* = 0.002; Figure 3E).

Furthermore, the predictive value of prognosis of the ICI score was validated in the total BLCA cohort (*n* = 584), including TCGA-BLCA (*n* = 406), GSE13507 (*n* = 61), GSE31684 (*n* = 74), and E-MTAB-1803 (*n* = 43). Survival analysis showed that the prognosis of patients in the high ICI score group was better than that in the low ICI score group (log-rank test, *p* = 0.017; Supplementary Figure 5A). In addition, we validated the ICI score in a completely independent external cohort (GSE93527). Survival analysis revealed that the prognosis of patients in the high ICI score group was better than that in the low ICI score group (log-rank test, *p* = 0.01; Supplementary Figure 5B). Finally, we validated the results on cell lines of bladder cancer. We calculated the ICI score for each cell line and grouped them into two groups by the median scores. Subsequently, we conducted differential expression analysis between the two groups and obtained 886 differentially expressed genes (| log2FC| > 1.65 and *p*-value < 0.05). Finally, we conducted hierarchical clustering of cell lines based on these differentially expressed genes, and finally obtained two classes, class 1 and class 2 (Supplementary Figure 5C). We analyzed and compared the ICI score of the two classes, and the results showed that the ICI score of class 2 was significantly higher than that of class 1 (Wilcoxon test, *p* = 0.019; Supplementary Figure 5D). It indicated that the differentially expressed genes defined were related to the ICI score, and also verified the prognostic effect of ICI score from another aspect.

### The Relationship Between the ICI Score and Somatic Mutation and Copy Number Variation

**FIGURE 4:**
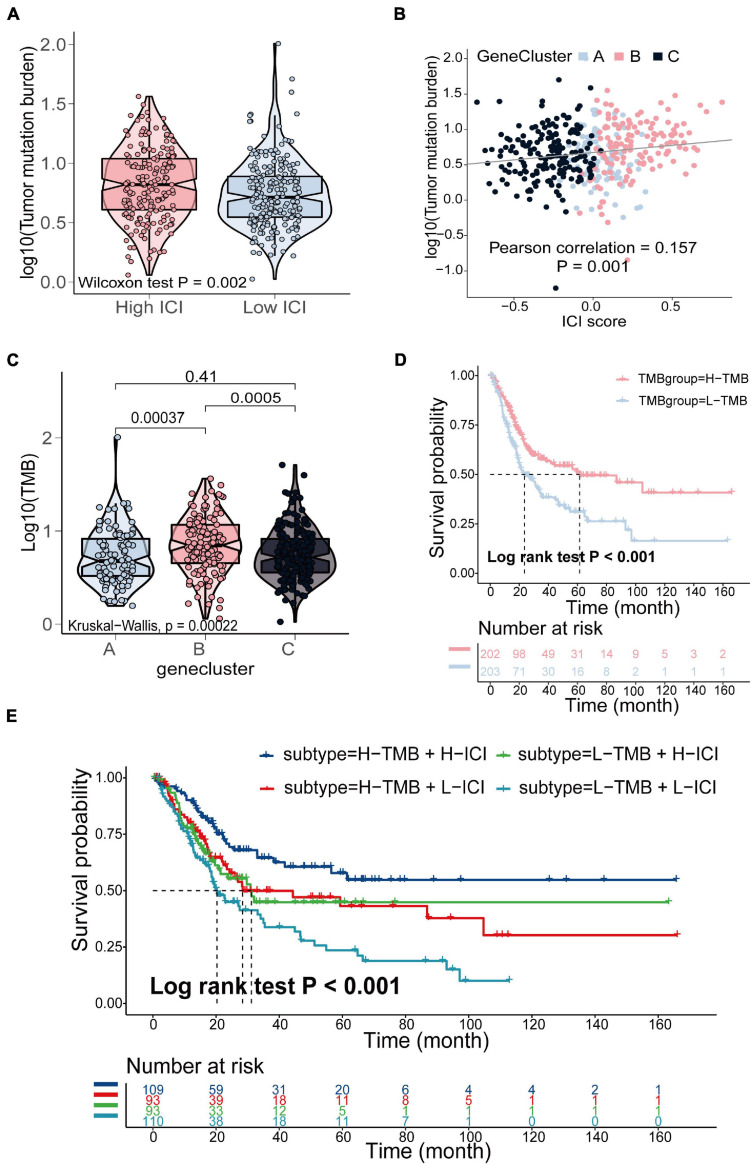
The relationship between the ICI score and somatic mutation in the TCGA- BLCA cohort. **(A)** TMB difference in the high and low ICI score subgroups (Wilcoxon test, *p* = 0.002). **(B)** Pearson correlation analysis between ICI score and mutation load (Pearson correlation coefficient = 0.157, *p* = 0.001). **(C)** The comparison of TMB among three gene clusters (Kruskal–Wallis test, *p* = 0.0002). **(D)** Kaplan–Meier curves for high and low TMB groups of (log-rank test, *p* < 0.001). **(E)** Kaplan–Meier curves for patients stratified by both TMB and ICI score (log-rank test, *p* < 0.001).

**FIGURE 5:**
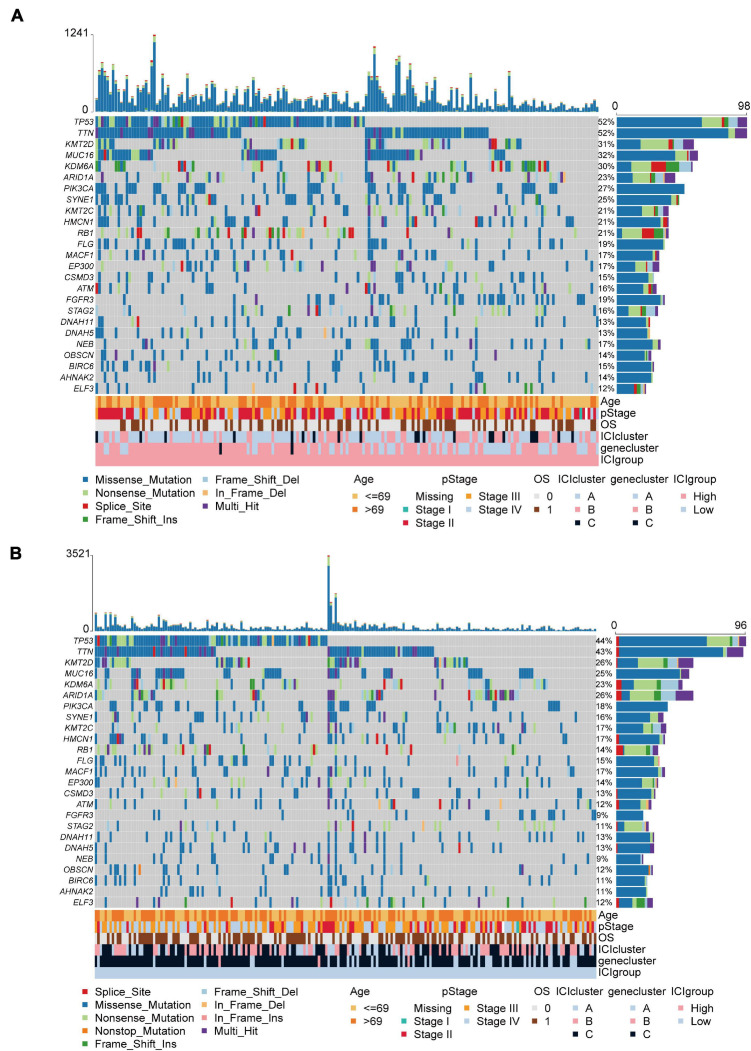
The oncoPrint in the high **(A)** and low ICI score group **(B)**.

The TMB is a crucial biomarker in cancer immunotherapy (Chan et al., 2019). Studies show that a higher TMB is correlated with stronger antitumor immunity (Rooney et al., 2015). Our analysis revealed that patients in the high ICI score group exhibited significantly higher TMB than those in the low ICI score group (Wilcoxon test, *p* = 0.002; Figure 4A). Pearson correlation analysis confirmed that the ICI score was significantly and positively correlated with TMB (Pearson correlation coefficient = 0.157, *p* = 0.001; Figure 4B). In addition, we found significant differences in TMB between the three gene clusters. The patients in gene cluster B exhibited significantly higher TMB than those in gene cluster A and C (Kruskal–Wallis test, *p* = 0.0002; Figure 4C). Next, we divided patients into high and low TMB subgroups according to the median TMB value. Survival analysis showed that patients in the high TMB subgroup presented better OS than those in the low TMB subgroup (log-rank test, *p* < 0.001; Figure 4D). To evaluate the synergistic effect of ICI score and TMB in bladder cancer, we further classified patients into four subgroups: high TMB + high ICI score, high TMB + low ICI score, low TMB + high ICI score, and low TMB + low ICI score. The stratified analysis revealed that TMB status did not affect the prediction of ICI score, and the high ICI score group exhibited significantly better survival in both the high and low TMB subgroups (log-rank test, *p* < 0.001; Figure 4E). We also evaluated somatic variants of driver genes between the low and high ICI groups, which were determined using the R package “maftools.” The top 25 driver genes with the highest mutation frequency in the high and low ICI score subgroups were analyzed (Figures 5A,B). The results revealed that the mutation frequency of FGFR3, NEB, PIK3CA, and SYNE1 in the high ICI score group was significantly higher than that in the low ICI score group, which provides some new ideas regarding the association between ICI and somatic mutation in immune checkpoint inhibitor therapy (Table 1).

**TABLE 1:** The association between ICI score and somatic variants.

Hugo_Symbol	High score		Low score		*p*-value
	Mutated samples	Mutation frequency	Mutated samples	Mutation frequency	
FGFR3	36	19%	20	9%	0.0042
NEB	33	17%	20	9%	0.0139
PIK3CA	51	27%	38	18%	0.0214
SYNE1	47	25%	35	16%	0.0287
KDM6A	57	30%	49	23%	0.0829
TTN	98	52%	94	43%	0.0858
RB1	39	21%	31	14%	0.0912
MUC16	61	32%	54	25%	0.0993
STAG2	31	16%	24	11%	0.1167
TP53	98	52%	96	44%	0.1256
BIRC6	28	15%	23	11%	0.2011
FLG	36	19%	32	15%	0.2470
ATM	30	16%	26	12%	0.2567
AHNAK2	27	14%	23	11%	0.2595
HMCN1	39	21%	36	17%	0.2948
KMT2D	58	31%	57	26%	0.3241
KMT2C	39	21%	37	17%	0.3557
EP300	32	17%	31	14%	0.4627
ARID1A	44	23%	57	26%	0.4874
CSMD3	28	15%	29	13%	0.5774
OBSCN	26	14%	27	12%	0.6950
ELF3	22	12%	27	12%	0.8045
DNAH11	25	13%	28	13%	0.9229
DNAH5	25	13%	28	13%	0.9229
MACF1	32	17%	36	17%	0.9268

**FIGURE 6:**
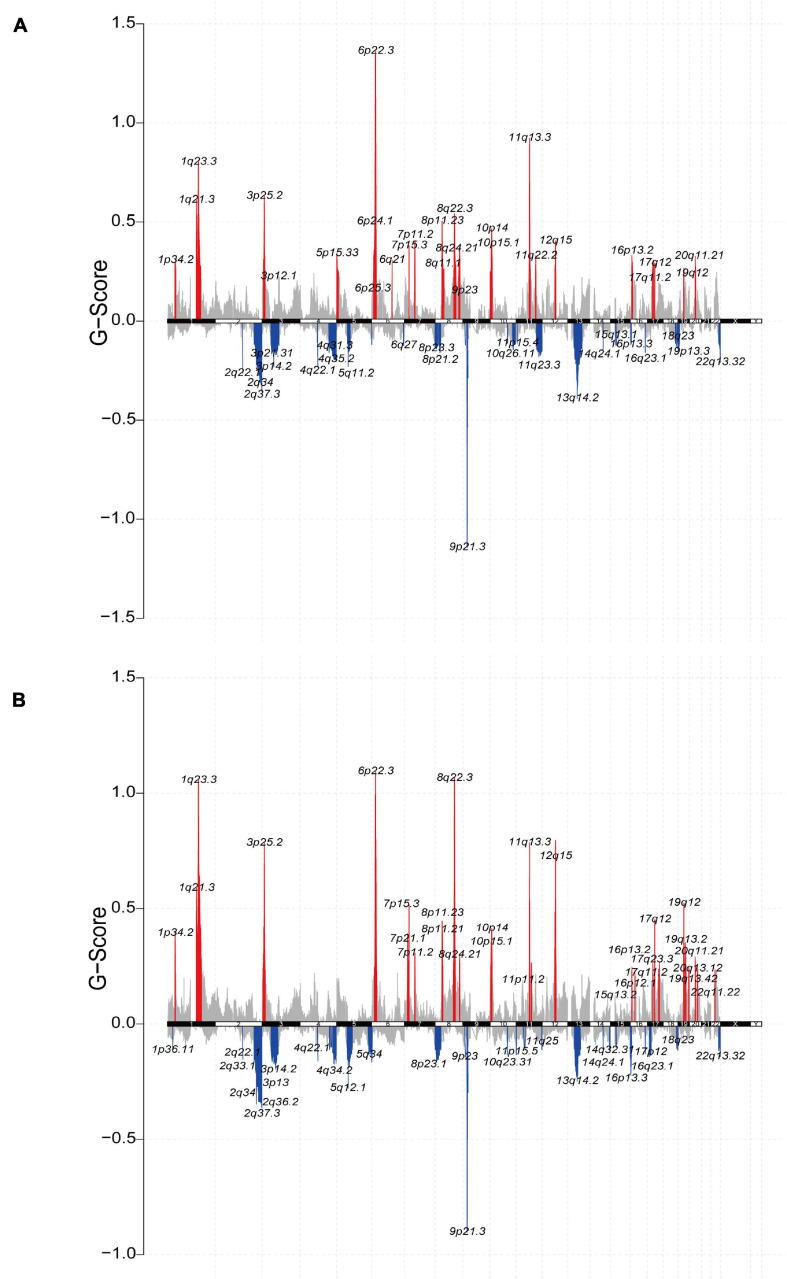
The relationship between the ICI score and copy number variation in the TCGA-BLCA cohort. **(A,B)** The visualization of the copy number variation analysis based on GISTIC2.0 for high **(A)** and low **(B)** ICI score groups.

Furthermore, we analyzed the relationship between the ICI score and copy number variation. GISTIC2.0 was used to analyze the copy number variation in the high and low ICI score subgroups (Mermel et al., 2011). The results were visualized using the R package “maftools” (Figures 6A,B). Significantly amplified regions in the high ICI score subgroup included 1q21.3, 1p34.2, 5p15.33, 6p24.1, etc. Significant amplified in the low ICI score subgroup included 3p25.2, 4q13.3, 6q23.3, 8p11.23, etc. In addition, we identified DEGs between the high and low ICI score groups, including 1140 genes that were highly expressed in the high ICI score group and 3419 genes that were highly expressed in the low ICI score group (| log2FC| > log2(1.2) and *p*adj < 0.05). And then we mapped the chromosomal locations of those genes. We performed Pearson correlation analysis between the expression of these genes and the copy number of the region in which the genes are located. According to the *r*-value, the correlation information of the first 24 genes in the high and low ICI score groups was plotted (Supplementary Figures 6A,B). For example, the expression of gene CD274 was positively correlated with the copy number of its region “9p24.1,” and the correlation coefficient was 0.525 (Supplementary Figure 6A). Therefore, the high expression of this gene is significantly correlated with the copy number of its region. Detailed correlation information of these genes is shown in Supplementary Table 7.

### The Relationship Between the ICI Score and Antitumor Immunity

**FIGURE 7:**
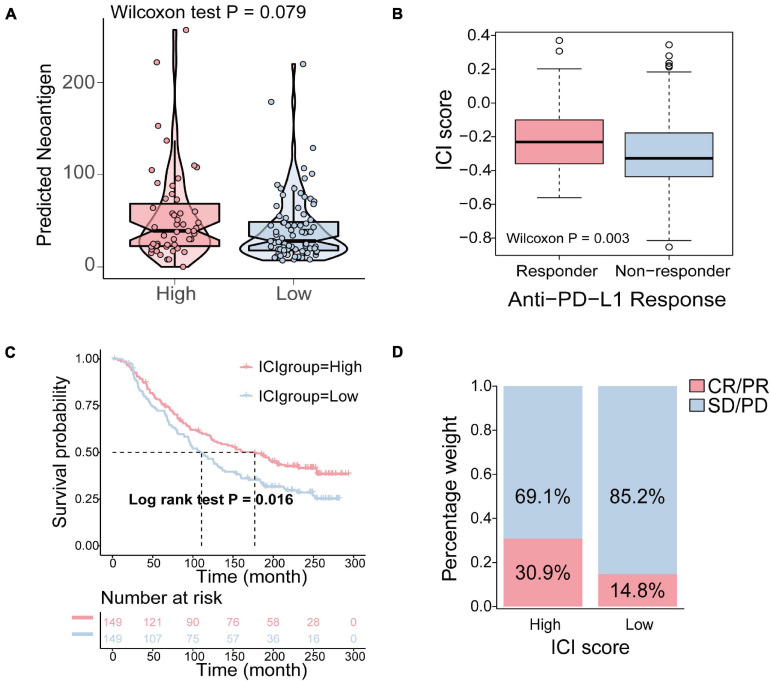
The relationship between the ICI score and antitumor immunity and the role of ICI pattern in anti-PD-L1 immunotherapy. **(A)** The difference of the number of neoantigens in the high and low ICI score groups (Wilcoxon test, *p* = 0.079). **(B)** The comparison of the ICI score in different anti-PD-1 clinical response statuses (Wilcoxon test, *p* = 0.003). **(C)** Kaplan–Meier curves for patients in the high and low ICI score group in the IMvigor210 cohort (log-rank test, *p* = 0.016). **(D)** The rate of clinical response to anti-PD-L1 immunotherapy in the high and low ICI score groups in the IMvigor210 cohort (CR, complete response; PR, partial response; SD, stable disease; PD, progressive disease).

To determine how the ICI score enhanced the immunogenicity of bladder cancer and activated antitumor immunity, we compared the expression of 74 immune-related genes between the high ICI score and low ICI score groups. The 74 immune-related genes were obtained from a published article (Yi et al., 2020). The results revealed that the expression of 53 immune-related genes was significantly different between the high and low ICI score group (Supplementary Figure 7). The detailed information of 74 immune-related genes is shown in Supplementary Table 8. Tumor neoantigens are abnormal proteins or antigens produced by mutations in the genes of tumor cells that are recognized by immune cells and can activate the immune system (Lennerz et al., 2005; Zhou et al., 2005). Neoantigens are a key target of cancer immunotherapy (Gubin et al., 2015). The feasibility of developing personalized immunotherapies based on neoantigens has been demonstrated (Nielsen et al., 2007; Mahesh, 2014). Therefore, it is necessary to explore the relationship between neoantigens and ICI score. The number of predicted neoantigens of some TCGA-BLCA samples was obtained from a published article (Rooney et al., 2015). The results demonstrated that the number of neoantigens in the high ICI score group was higher than that in the low ICI score group, but the differences were not statistically significant (Wilcoxon test, *p* = 0.079; Figure 7A).

### The Role of ICI Pattern in Anti-PD-L1 Immunotherapy

Immune checkpoints are a series of molecules that are expressed on immune cells and regulate the degree of immune activation. They play an important role in preventing the occurrence of autoimmune action (Pardoll, 2012). Immunotherapy induced by the blockade of PD-L1 and PD-1 is undoubtedly a breakthrough in cancer treatment (Sharma et al., 2011; Hinrichs and Rosenberg, 2014). As a result, based on an anti-PD-L1 immunotherapy cohort, IMvigor210, we investigated to determine whether the ICI score predicts patients’ responses to immune checkpoint inhibitors. Similarly, patients were classified into two groups as the high and low ICI score groups by the median score. We found that higher ICI score were associated with objective responses to anti-PD-L1 treatment, and patients with responses to anti-PD-L1 treatment exhibited higher ICI score in the Imvigor210 cohort (Wilcoxon test, *p* = 0.003; Figure 7B). Patients with high ICI score exhibited significantly prolonged OS (log-rank test, *p* = 0.016; Figure 7C). In addition, patients in the high ICI score group exhibited a significantly higher objective response rate (CR/PR) to anti-PD-L1 immunotherapy than those in the low ICI score group (Figure 7D). In conclusion, these results indicated that the ICI score is significantly associated with anti-PD-L1 immunotherapy responses and that the established ICI score can help predict the anti-PD-L1 immunotherapy response.

**FIGURE 8:**
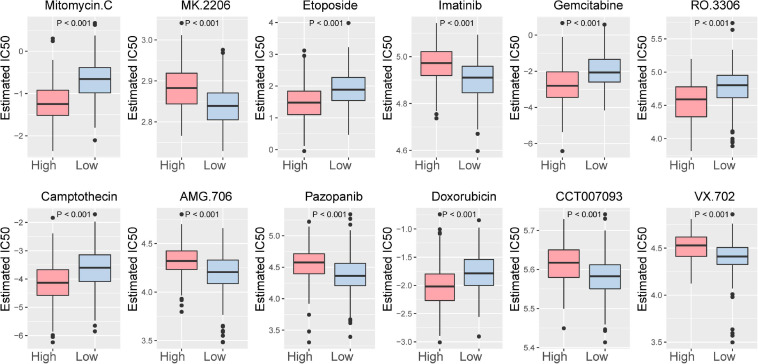
The first 12 drugs with different responses between the high ICI score subgroup and low ICI score subgroup.

In addition, the relationship between the ICI score and the sensitivity of drug therapy was examined. We analyzed the effect of the ICI score on the sensitivity of bladder cancer cells to drug therapy in the TCGA-BLCA cohort based on Genomics of Drug Sensitivity in Cancer (GDSC)^7^. Among them, 92 drugs exhibited significantly different responses between the high and low ICI score groups (Supplementary Figure 7). The high ICI score group exhibited increased bladder cancer sensitivity to 50 drugs. According to the *p*-value, the first 12 drugs with different responses were plotted (Figure 8).

## Discussion

Immunotherapy has evolved in recent years and has been proven to treat a variety of cancers, including melanoma, non-small cell lung cancer, kidney cancer and prostate cancer (Del Paggio, 2018). It functions by blocking PD-L1 and PD-1. PD-1 is an inhibitory receptor expressed on the surface of activated T cells with two ligands, PD-L1 and PD-L2. PD-L1 is generally widely expressed on the surface of epithelial cells, endothelial cells, and tumor cells. PD-1/PD-L1 inhibitors have been successful in the treatment of melanoma and therefore are also being studied in urothelial carcinoma. A high level of PD-L1 expression was found in the TME of urothelial carcinoma, which increases the recognition of exogenous antigens by the host immune system (Inman et al., 2007; Chen and Mellman, 2013). A 2016 study reported that a PD-L1 monoclonal antibody, durvalumab (MEDI4736), exhibited some clinical efficacy in patients with bladder urothelial carcinoma receiving multiline therapy (Massard et al., 2016). However, studies have shown that immunotherapy is effective in only a small number of patients (Julie and Scott, 2012; Christofi et al., 2019). Therefore, new therapeutic markers are needed to identify the subgroup of patients who are suitable for immunotherapy. In this study, we identified three ICI subtypes based on ICI and then identified three gene clusters based on the DEGs between the ICI subtypes. In addition, we developed a method to quantify the tumor immune environment of individual tumors. Our results suggested that the ICI score is a valid prognostic predictor for evaluating immunotherapy response and provides a worthy reference for the immunotherapy of bladder cancer.

The TME that surrounds tumor cells is composed of tumor-infiltrating immune cells (TIICs), mesenchymal cells, endothelial cells, inflammatory mediators, and ECM molecules (Hanahan and Coussens, 2012). A large number of studies have shown that the TME has significant effects on tumor growth and development, therapeutic resistance and clinical outcome (Wu and Dai, 2017; Peltanova et al., 2019; Baghban et al., 2020). In this study, we identified three distinct ICI subtypes based on the ICI pattern of 584 bladder cancer samples. The characteristics of the TME and the proportions of 22 tumor immune infiltration cells were significantly different among the three ICI subtypes. This suggests the critical role of ICI in cancer progression. In detail, a previous study demonstrated that the functions of B cells are different in different types of cancer (Liu et al., 2018). B cells can be activated by tumor cells and then secrete immunoglobulin to inhibit tumor growth (Li et al., 2009). Some studies have shown that B cell infiltration promotes tumor invasion and metastasis in bladder cancer (Ou et al., 2015). In our study, there was no significant difference in the infiltration of naive B cells among the three ICI subtypes, while the infiltration level of memory B cells in ICI subtype C with the poorest prognosis was significantly higher than that in ICI subtypes A and B. This indicates that B cell infiltration promotes tumor invasion and metastasis in bladder cancer, consistent with a previous study (Ou et al., 2015). Memory T cells, effector T cells and T cell differentiation play an important role in immune defense (Li et al., 2020). T cells can be classified into CD4+ and CD8+ T cells, and CD4+ T cells can further differentiate into regulatory T cells (Tregs) and follicular helper T cells (Tfhs). Tregs are responsible for maintaining the balance of immune responses and preventing excessive immune responses, and they are thought to be involved in the escape of tumors from the host’s immune system in cancer (Whiteside, 2014). They have also been shown to have a positive prognostic effect on bladder cancer (Winerdal et al., 2018). Tfhs are thought to play a key role in coordinating humoral-related immune responses, and the abnormal regulatory behavior of Tfhs contributes to autoimmune diseases, primary immunodeficiency and acquired immunodeficiency (Jia et al., 2015). In addition, studies have shown that high expression of Tfh-related genes in colorectal cancer and breast cancer is associated with a good prognosis (Shi et al., 2018). In our study, ICI subtype A, with a better prognosis, exhibited higher immune infiltration of Treg and Tfh cells. These results imply that Tregs and Tfh cells play a positive role during the development of bladder cancer, which is consistent with previous studies (Shi et al., 2018; Winerdal et al., 2018). Macrophages, including M1 and M2 macrophages, are an important part of innate and adaptive immunity (Hao et al., 2012). Studies have shown that M1 macrophages have the opposite effect as M2 macrophages. M1 macrophages participate in a positive immune response and play the role of immune surveillance by secreting proinflammatory cytokines and chemokines and presenting antigens. M2 macrophages only have a weak antigen-presenting ability and play an important role in immune regulation by secreting inhibitory cytokines to downregulate the immune response (Chanmee et al., 2014). In this study, we found that infiltration of M1 macrophages was higher in ICI subtype A with a better prognosis, while the infiltration level of M2 macrophages was higher in ICI subtype C with a poorer prognosis, consistent with known findings.

Studies have shown that only a small number of patients respond to immunotherapy, which suggests that the immune phenotype cannot completely or accurately predict the response of patients to immunotherapy (Julie and Scott, 2012; Christofi et al., 2019). Therefore, we identified three gene clusters based on DEGs between three ICI subtypes. Gene cluster B, with the highest immune score, exhibited higher infiltration of plasma cells, CD8+ T cells and activated CD4+ T cells, and the expression levels of PD1 and PD-L1 were higher in this cluster, presenting an immune-hot phenotype. We speculate that patients in this cluster might benefit from immunotherapy. Gene cluster A had the lowest immune and stroma scores, and the infiltration level of immune-associated cells was decreased, which suggests an immune-cold phenotype. A good prognosis in this cluster may be related to the high immune infiltration of naive CD4+ T cells, which can rapidly differentiate into effector, regulatory, or memory T cells activated by antigen-presenting cells.

Considering the individual heterogeneity of the TME, it is necessary to quantify the ICI pattern of individual tumors (Runa et al., 2017). For that, we established the ICI score to evaluate the degree of individual patient immune infiltration in bladder cancer. Our analysis suggested that patients in the high ICI score group had a favorable prognosis and higher expression of checkpoint-related and immune activity-related genes. The GSEA results showed that ALLOGRAFT_REJECTION, INTERFERON_ALPHA_RESPONSE and INTERFERON_GAM MA_RESPONSE were significantly enriched in the high ICI score group, while ANGIOGENESIS, COAGULATION and EPITHELIAL_MESENCHYMAL_TRANSITION were enriched in the low ICI score group. ALLOGRAFT_REJECTION involves cytokine-cytokine receptor interactions and IL-12-mediated signal-related pathways, and upregulation of this gene set is associated with the activation of the acute immune response. Interferon is a cytokine that can trigger protective defenses of the immune system, activate immune cells, upregulate antigen presentation and prevent viral replication (Parkin and Cohen, 2001). A previous study demonstrated that an improvement in survival was observed when interferon was administered to patients with bladder cancer (Flávia et al., 2018). Conversely, tumor angiogenesis is a prerequisite for tumor growth and metastasis and is associated with reduced survival in bladder cancer (Kong et al., 2005; Agrawal et al., 2011). Increased expression of coagulation factors was observed in cancer patients, and coagulation factors may promote migration and invasion by transforming macrophages into tumor-associated macrophages in gastric cancer (Ma et al., 2011). Epithelial-mesenchymal transition (EMT) is a process in which epithelial cells with polarity are transformed into transitional mesenchymal cells and acquire the ability to invade and migrate, which exists in multiple physiological and pathological processes of the human body. EMT is closely related to the invasion and metastasis of tumor cells (Thiery, 2003).

In addition, studies have shown that increased TMB and somatic mutation rates are correlated with stronger antitumor immunity (Rooney et al., 2015). Therefore, it is necessary to explore the relationship between TMB and ICI score. Survival analysis demonstrated that the high TMB group had a better prognosis than the low TMB group. Our analysis also showed a significantly positive correlation between ICI score and TMB with a correlation coefficient of 0.157. These results were consistent with previous studies. Stratification analysis revealed that the ICI score was a potent biomarker of prognosis independent of TMB. In addition, studies have shown that FGFR3 mutations occur in 50% of primary bladder tumors and are associated with a favorable prognosis (van Rhijn et al., 2003; Oers et al., 2009; Saing et al., 2018). PIK3CA mutations were also associated with improved outcomes (Kim et al., 2015). Our results revealed that the mutation frequency of FGFR3 and PIK3CA in the high ICI score subgroup with better prognosis was significantly higher than that in the low ICI score subgroup. Finally, the prognostic value of the ICI score was validated in all BLCA cohorts.

In short, we analyzed the ICI pattern, providing a clear view of the antitumor immune or protumor immune response in bladder cancer. We found that the difference in ICI patterns was correlated with tumor heterogeneity and treatment complexity. Thus, systematic evaluation of tumor ICI patterns in this study has crucial clinical implications. Moreover, our results provide new ideas for improving patient clinical response to immunotherapy and promoting individualized tumor immunotherapy in the future.

## Conclusion

In this study, we identified three ICI subtypes based on ICI and then identified three gene clusters based on the DEGs among the ICI subtypes. In addition, we developed a method to quantify the tumor immune environment of individual tumors. Our results suggested that the ICI score is a valid prognostic biomarker and predictor for evaluating immunotherapy response, providing new ideas for improving patients’ response to immunotherapy and promoting individualized tumor immunotherapy in the future.

## Data Availability Statement

Raw data for this study were generated at the TCGA database with the cancer type of BLCA. The datasets used and/or analyzed during the current study are available from the GEO database (GSE13507 and GSE93527) and the Array Express database (GSE31684 and E-MTAB-1803). Derived data supporting the findings are available from the corresponding author (FY) on reasonable request.

## Author Contributions

FY and XL designed and guided the work. MH and LL participated in data collecting, data processing, program implementation, and manuscript writing. JZ and TJ contributed to statistical analysis. YC, LX, and WC contributed to manuscript writing and article publishment. JM revised the manuscript critically. All authors provided critical advice for the final manuscript.

## Conflict of Interest

The authors declare that the research was conducted in the absence of any commercial or financial relationships that could be construed as a potential conflict of interest.

## Publisher’s Note

All claims expressed in this article are solely those of the authors and do not necessarily represent those of their affiliated organizations, or those of the publisher, the editors and the reviewers. Any product that may be evaluated in this article, or claim that may be made by its manufacturer, is not guaranteed or endorsed by the publisher.

## Footnotes

1
http://firebrowse.org/

2
https://www.cbioportal.org/

3
https://www.ncbi.nlm.nih.gov/geo/

4
https://www.ebi.ac.uk/arrayexpress/

5
http://www.gsea-msigdb.org/gsea/msigdb

6
https://depmap.org/portal/

7
https://www.cancerrxgene.org/
