# Supplementary material for: Identification of Immune-Related Subtypes and Characterization of Tumor Microenvironment Infiltration in Bladder Cancer
Source: Front Cell Dev Biol. 2021 Aug 31;9:723817. doi: 10.3389/fcell.2021.723817 (PMC8438153; doi:10.3389/fcell.2021.723817)
Supplement: Supplementary file 1 [file Data_Sheet_1.zip › Supplementary Materials/Supplementary_Figures.docx]

**Supplementary Figures**


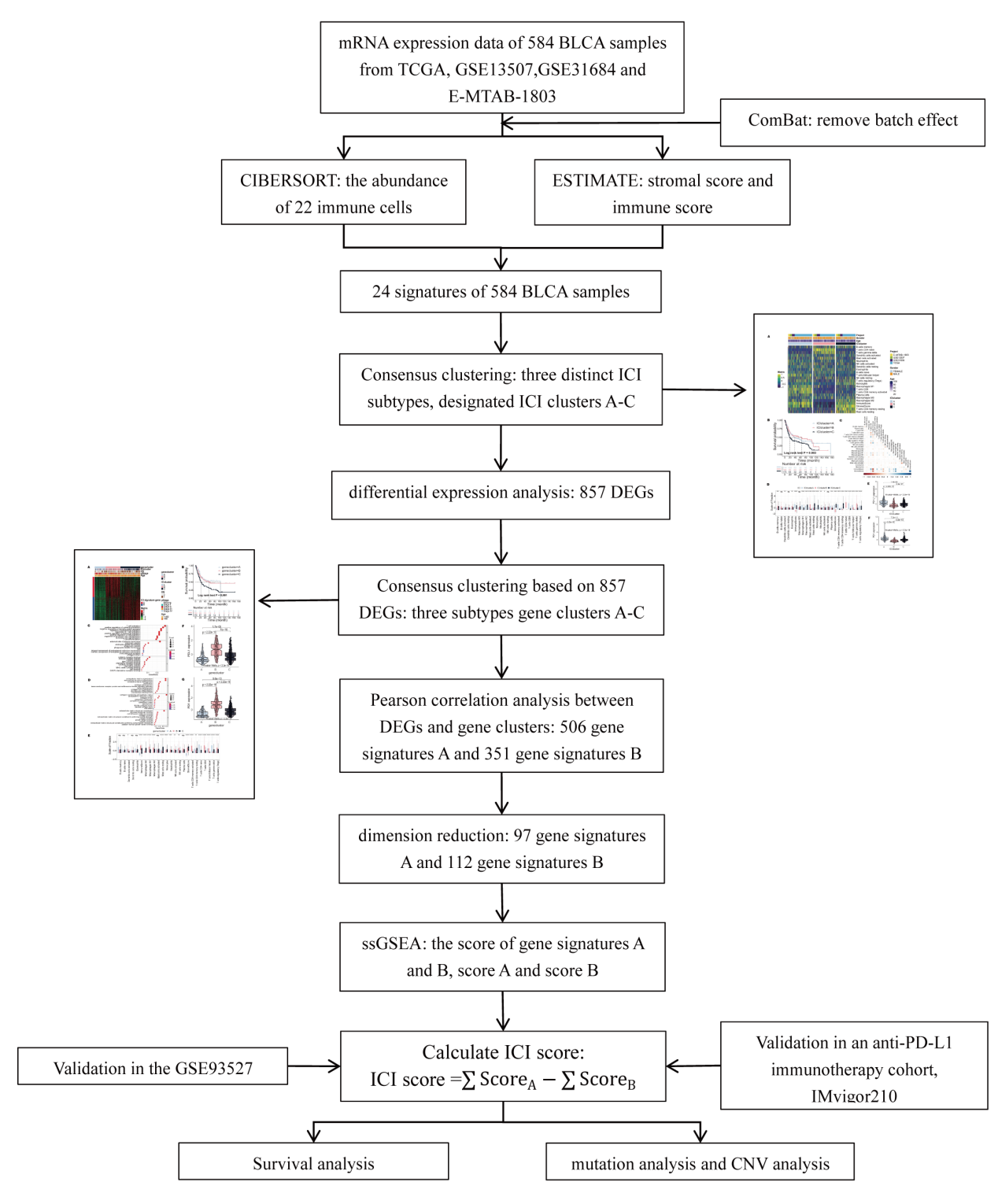


**Supplementary Figure S1.** The flow chart for construction of the ICI score.


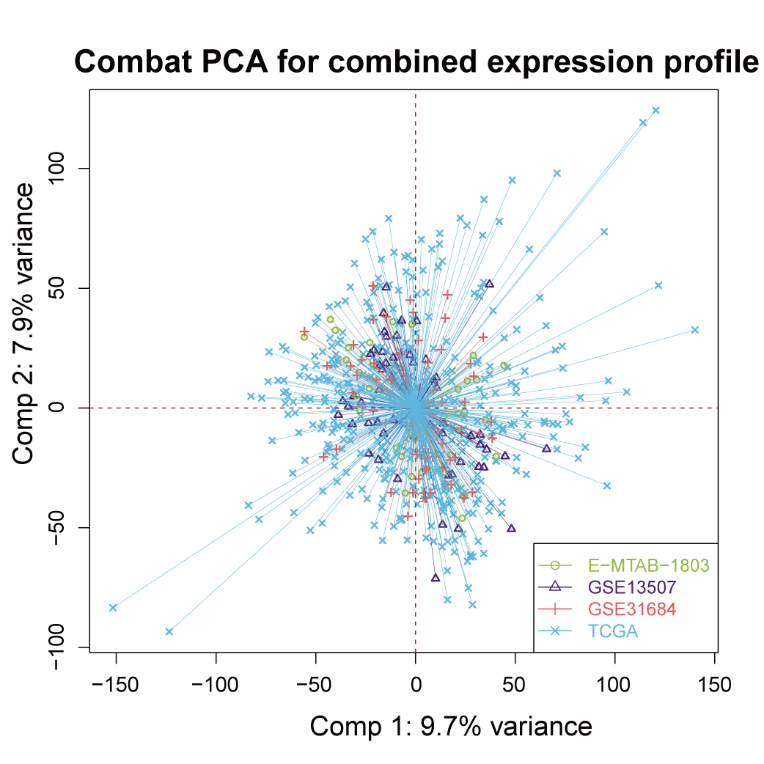


**Supplementary Figure S2.** The combat PCA for combined expression profile of four independent bladder cancer cohorts.


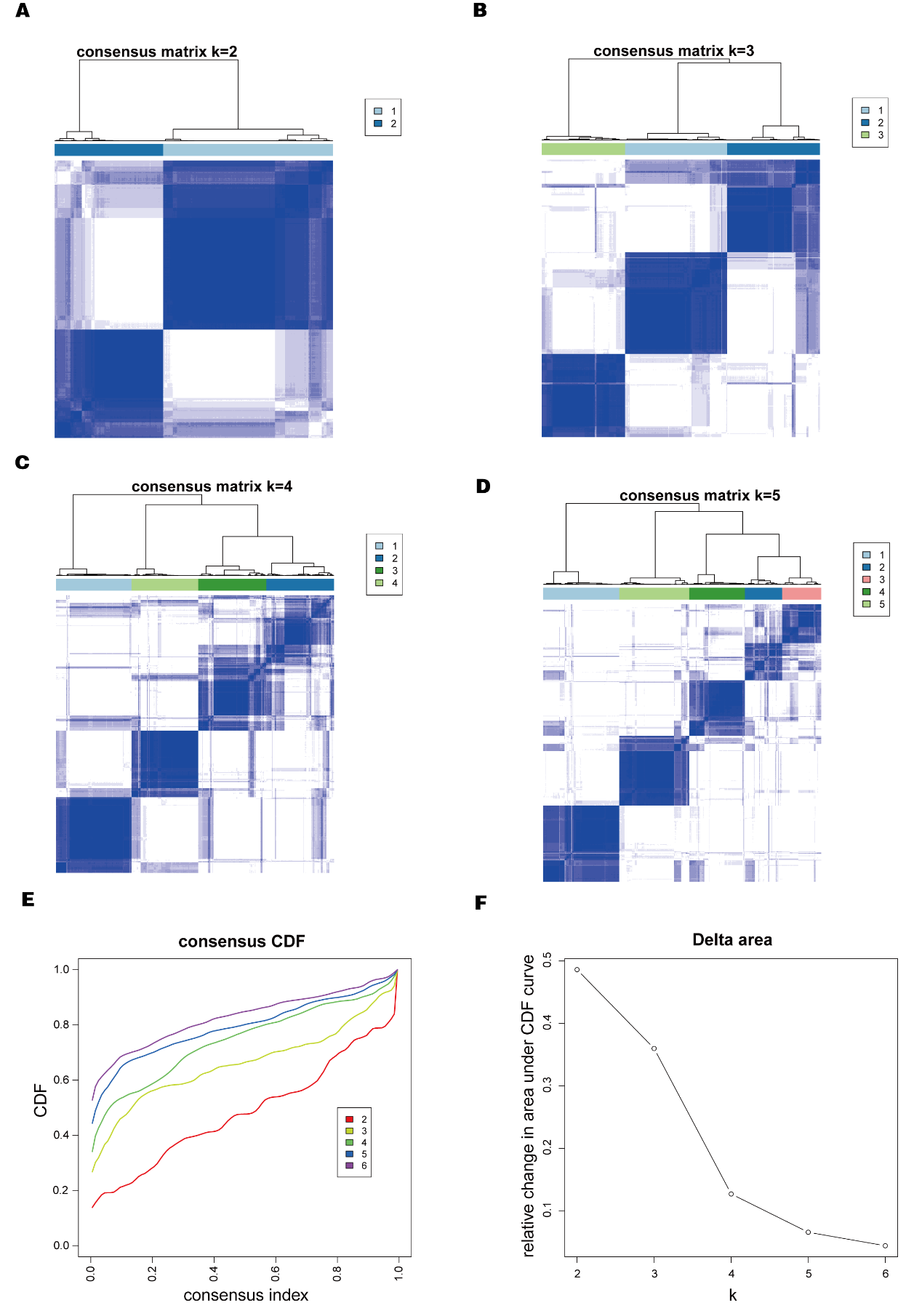


**Supplementary Figure S3.** Measuring consensus and determining the number of subtypes (optimal k) in all BLCA samples. **(A-D)** Heat map of the consensus matrix for (A) k = 2, (B) k = 3, (C) k = 4, and (D) k = 5. **(E)** Cumulative distribution function (CDF) curve of the consistency score for different subtype numbers (k = 2-6). **(F)** Delta area plot of the relative increase in cluster stability when k = 3.


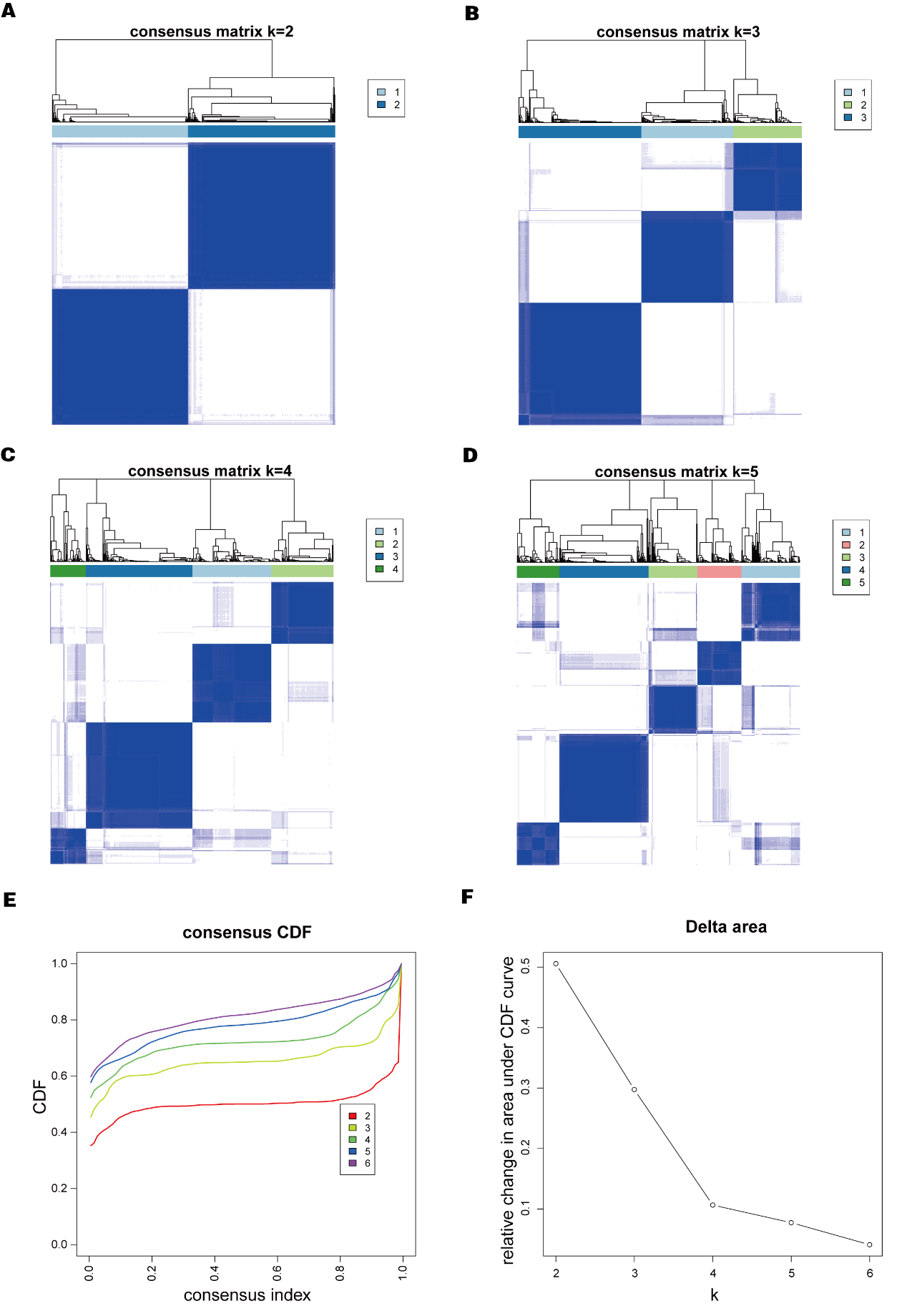


**Supplementary Figure S4.** Measuring consensus and determining the number of subtypes (optimal k) in the TCGA-BLCA cohort. **(A-D)** Heat map of the consensus matrix for (A) k = 2, (B) k = 3, (C) k = 4, and (D) k = 5. **(E)** Cumulative distribution function (CDF) curve of the consistency score for different subtype numbers (k = 2-6). **(F)** Delta area plot of the relative increase in cluster stability when k = 3.


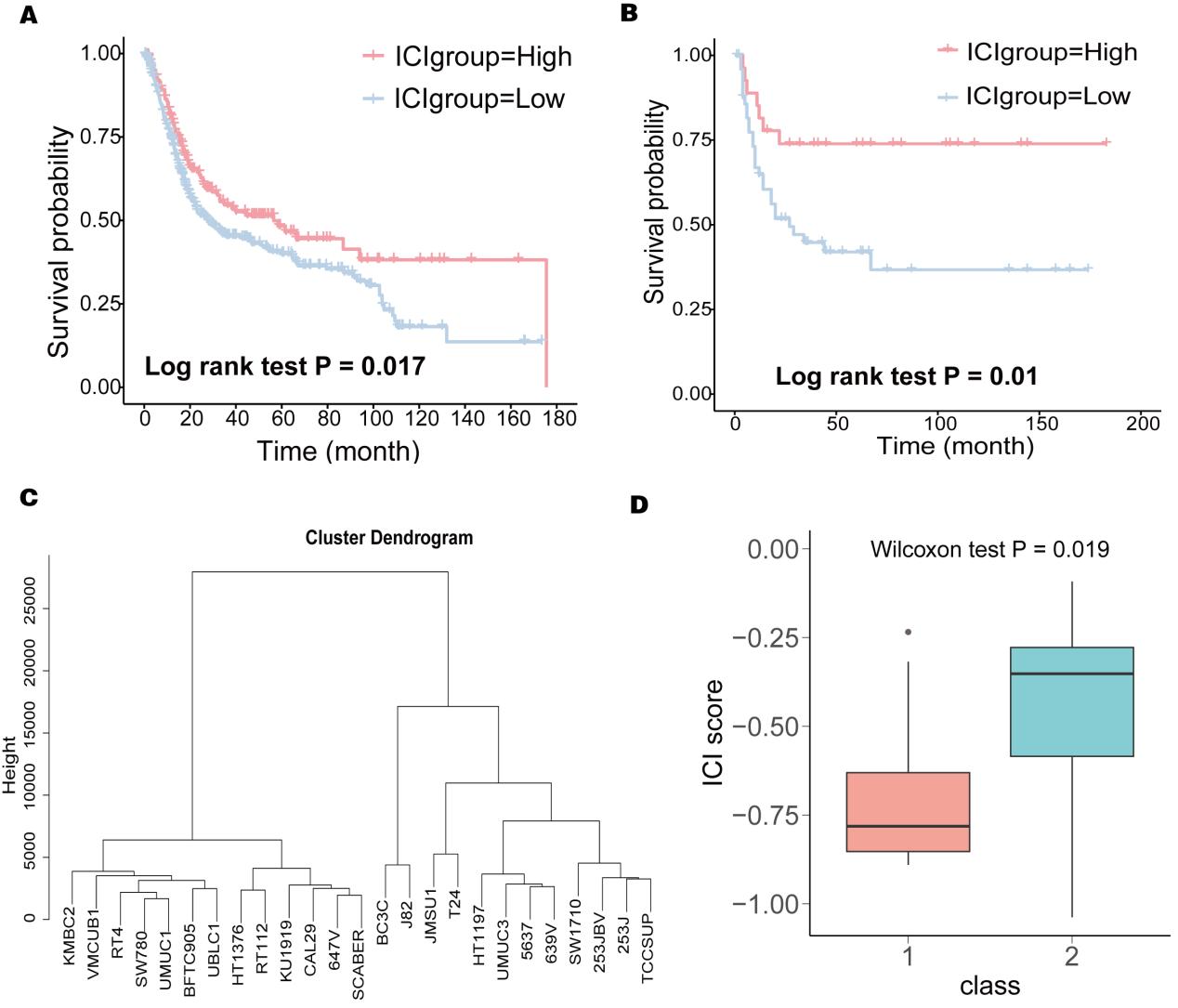


**Supplementary Figure S5.** Prognostic value of ICI score in validation cohorts. **(A)** Kaplan-Meier curves for patients with high and low ICI score in the total BLCA cohorts including TCGA-BLCA, GSE13507, GSE31684 and E-MTAB-1803. (log-rank test, p = 0.017). **(B)** Kaplan-Meier curves for patients with high and low ICI score in a completely independent external cohort: the GSE93527 cohort (log-rank test, p = 0.01). **(C)** Hierarchical clustering for cell lines in bladder cancer. **(D)** The ICI score difference in the two classes obtained by hierarchical clustering (Wilcoxon test, p = 0.019).


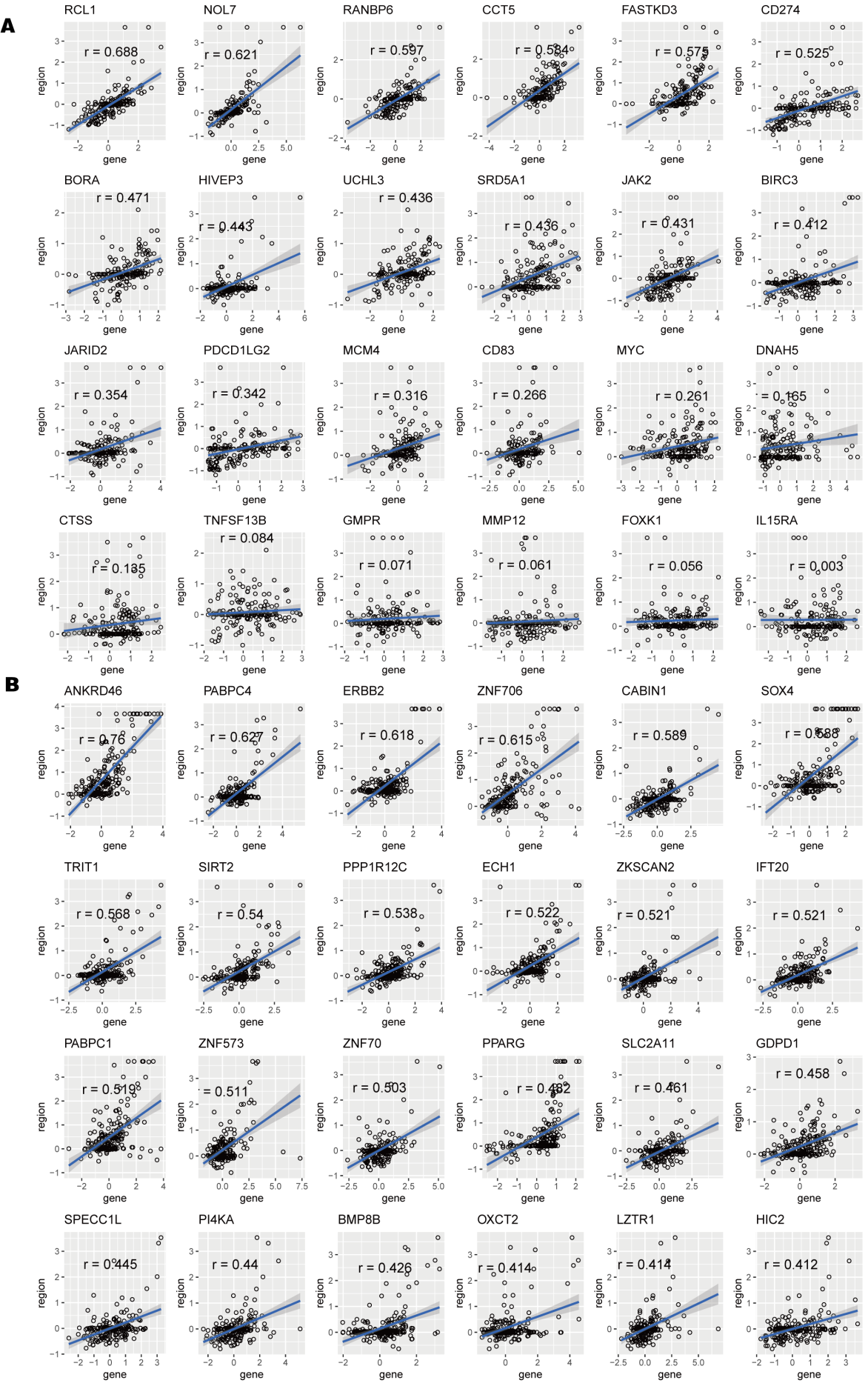


**Supplementary Figure S6.** Correlation analysis of differentially expressed genes with the copy number of the region in which the gene resides in the high (A) and low (B) ICI score group.


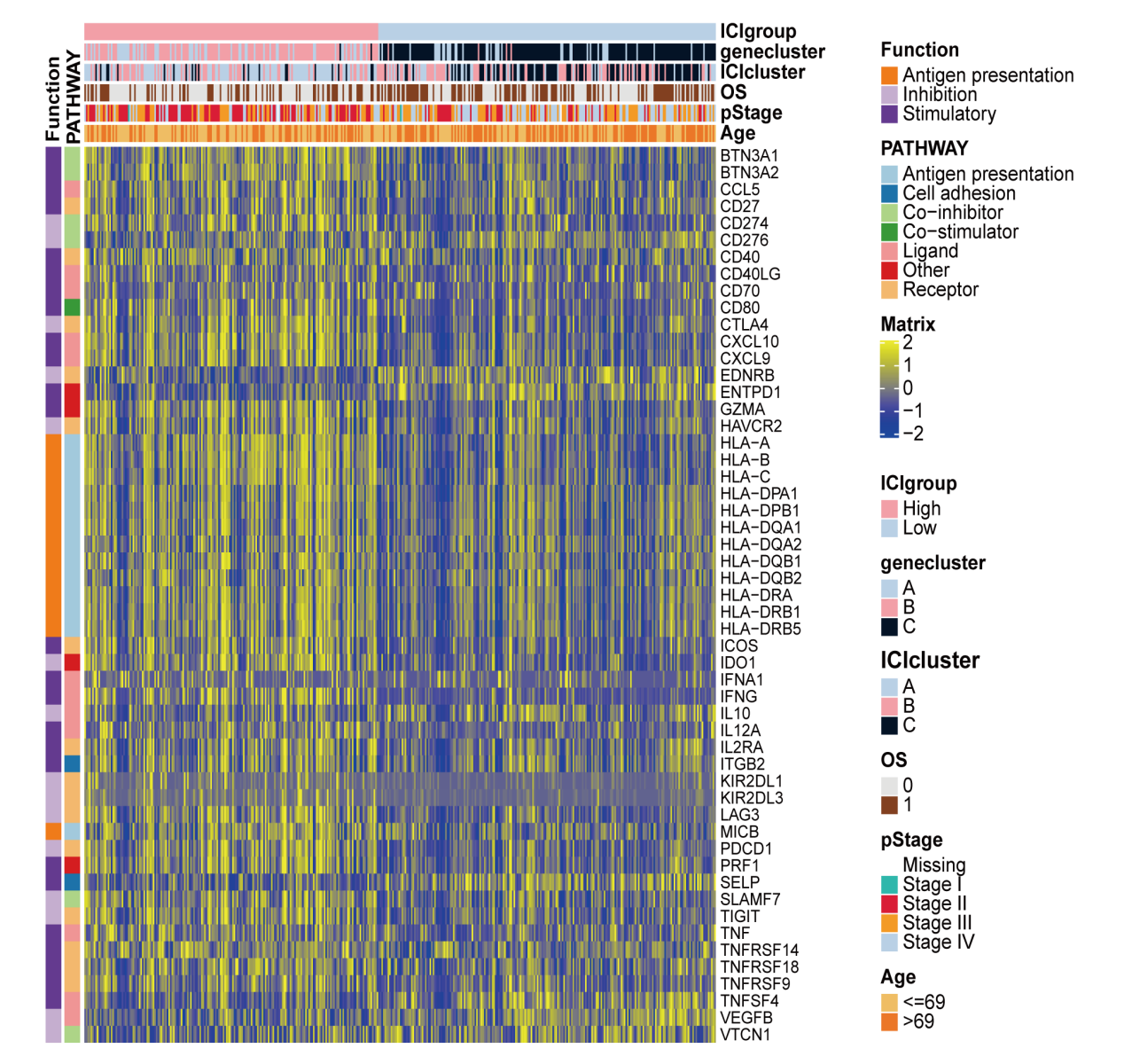


**Supplementary Figure S7.** Heatmap showing average changes in the expression levels of 53 immune-related genes between high ICI score subgroup and low ICI score subgroup in the TCGA-BLCA cohort.


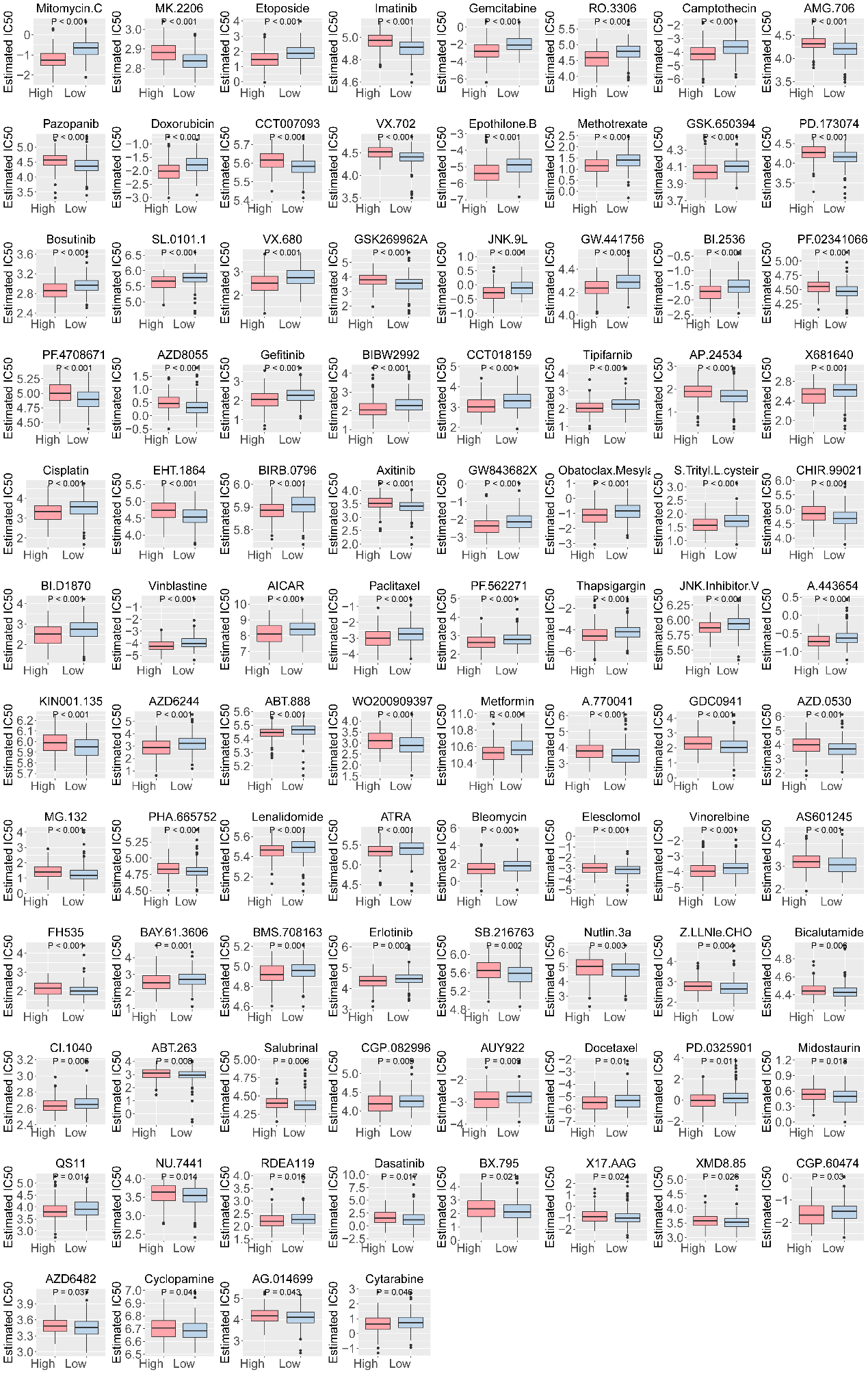


**Supplementary Figure S8.** Comparison of drug sensitivity between the high and low ICI score subgroup in the GDSC-bladder cancer cell line cohort.
